# Utilidade de Biomarcadores na Suspeita de Amiloidose Cardíaca: Oportunidade para Diagnóstico mais Frequente e Precoce

**DOI:** 10.36660/abc.20220437

**Published:** 2022-07-29

**Authors:** João Marcos Barbosa-Ferreira, Andreza Araújo de Oliveira

**Affiliations:** 1 Universidade Nilton Lins Manaus AM Brasil Universidade Nilton Lins, Manaus, AM – Brasil

**Keywords:** Amiloidose Cardíaca, Biomarcadores, NT-proBNP, Volume Sistólico, Insuficiência Cardíaca, Disfunção Ventricular, Diagnóstico por Imagem/métodos

A metanálise intitulada “Diagnostic Role of NT-proBNP in Patients with Cardiac Amyloidosis Involvement: A Meta-Analysis” nos traz uma importante revisão da utilidade da dosagem de NT-próBNP em pacientes com acometimento cardíaco pela amiloidose. A demonstração de boa sensibilidade e especificidade deste biomarcador reforça sua utilidade no diagnóstico da amiloidose cardíaca (AC).^1^

A amiloidose cardíaca tem sido cada vez mais diagnosticada, principalmente em pacientes com o fenótipo de insuficiência cardíaca com fração de ejeção preservada.^2^ Pouco mais da metade dos pacientes com sintomas de insuficiência cardíaca apresentam fração de ejeção preservada, especialmente os indivíduos idosos. Este achado habitualmente é considerado apenas como disfunção diastólica relacionada à idade e a comorbidades associadas. Porém, tal fator deve ser um dos sinais de alerta para o diagnóstico de AC, principalmente quando associado a níveis elevados de biomarcadores.^3^ É descrita uma grande variabilidade na frequência do diagnóstico de amiloidose cardíaca na população geral podendo chegar de 5 a 74% entre os diversos estudos.^1^ Esta variabilidade pode estar relacionada a fatores como baixa suspeição clínica ou dificuldades de acesso aos exames complementares necessários para o diagnóstico do acometimento cardíaco na amiloidose. O fluxograma diagnóstico em pacientes com suspeita de acometimento cardíaco pela amiloidose é baseado, principalmente, em exames de imagem.^4–6^ Estes exames, podem apresentar alto custo, como: cintilografia miocárdica, ecocardiograma com strain e ressonância magnética cardíaca e, muitas vezes, estão disponíveis apenas em centros de referência cardiológica tornando o diagnóstico da AC mais difícil e tardio.^3^ Além disto, é importante salientar que o diagnóstico tardio destes pacientes pode influenciar diretamente no prognóstico, por atrasar o início do tratamento, levando, por exemplo, a uma média de 6 meses de sobrevida após o desenvolvimento dos sintomas na forma AL da amiloidose.^4^

Portanto, principalmente em centros menos desenvolvidos, a AC ainda é subdiagnosticada, configurando um grave problema de saúde pública. Com isto, a utilização de exames não invasivos, de fácil acesso e baixo custo pode ser importante. Neste cenário, a dosagem de biomarcadores como o NT pro-BNP, troponina ou outros pode ser útil não só na avaliação inicial, mas também na avaliação prognóstica de pacientes com suspeita de amiloidose cardíaca. O NT pro-BNP se utiliza há vários anos no diagnóstico, acompanhamento clínico e prognóstico de pacientes com insuficiência cardíaca de uma forma geral.^7,8^ Estudos com NT-proBNP na AC tem evidenciado boa acurácia diagnóstica, inclusive fazendo parte da avaliação para estadiamento prognóstico da doença.^9,10^ Além da avaliação diagnóstica e prognóstica os biomarcadores podem também ser utilizados na avaliação da eficácia terapêutica destes pacientes, principalmente em pacientes hematológicos nos quais a quimioterapia pode ser cardiotóxica.^4^

É importante salientar que a amiloidose cardíaca é uma doença cada vez mais frequente, até pelo envelhecimento da população. No entanto, esta doença ainda é subdiagnosticada, principalmente em centros menos desenvolvidos ou onde exames de alto custo não são de fácil acesso à população que utiliza serviços públicos. Portanto, é necessária a organização de fluxogramas diagnósticos mais acessíveis à maior parte da população e a dosagem de biomarcadores como o NT-proBNP é muito útil neste cenário.
