# Supplementary material for: Utilidade de Biomarcadores na Suspeita de Amiloidose Cardíaca: Oportunidade para Diagnóstico mais Frequente e Precoce
Source: Arq Bras Cardiol. 2022 Jul 29;119(2):223–4. [Article in Portuguese] doi: 10.36660/abc.20220437 (PMC9363050; doi:10.36660/abc.20220437)
Supplement: Translation

# Translation: Utility of Biomarkers in Suspected Cardiac Amyloidosis: Opportunity for More Frequent and Early Diagnosis

**Authors:** João Marcos Barbosa-Ferreira, Andreza Araújo de Oliveira

**Affiliations:** 1 Manaus AM Brazil Universidade Nilton Lins, Manaus, AM – Brazil

**Keywords:** Amyloidosis, Cardiac, Biomarkers, NT-proBNP, Stroke Volume, Heart Failure, Ventricular Dysfunction, Diagnostic, Imaging/methods

The meta-analysis entitled “Diagnostic Role of NT-proBNP in Patients with Cardiac Amyloidosis Involvement: A Meta-Analysis” brings us an important review of the usefulness of NT-proBNP measurement in patients with cardiac involvement by amyloidosis. The demonstration of good sensitivity and specificity of this biomarker reinforces its usefulness in the diagnosis of cardiac amyloidosis (CA).^1^

Cardiac amyloidosis has been increasingly diagnosed, especially in patients with the heart failure phenotype of preserved ejection fraction.^2^ Just over half of the patients with symptoms of heart failure have preserved ejection fraction, especially elderly individuals. This finding is usually considered only as age-related diastolic dysfunction and associated comorbidities. However, this factor should be one of the warning signs for diagnosing CA, especially when associated with high levels of biomarkers.^3^ A great variability in the frequency of diagnosis of CA in the general population has been described, ranging from 5 to 74% between the various studies.^1^ This variability may be related to factors such as low clinical suspicion or difficulties accessing complementary exams necessary for diagnosing cardiac involvement in amyloidosis. The diagnostic flowchart in patients with suspected cardiac involvement by amyloidosis is mainly based on imaging tests.^4–6^ These tests can be expensive, such as myocardial scintigraphy, strain echocardiography and cardiac magnetic resonance, and, often, they are only available at cardiology referral centers, making the diagnosis of CA more difficult and delayed.^3^ In addition, it is important to note that the late diagnosis of these patients can directly influence the prognosis by delaying the start of treatment, leading to a median of 6 months of survival after the development of symptoms in the AL form of amyloidosis for example.^4^

Therefore, especially in less developed centers, CA is still underdiagnosed, configuring a serious public health problem. With this, the use of non-invasive, easily accessible and low-cost exams can be important. In this scenario, the measurement of biomarkers such as NT pro-BNP, troponin or others can be useful not only in the initial evaluation but also in the prognostic evaluation of patients with suspected cardiac amyloidosis. NT pro-BNP has been used for several years in the diagnosis, clinical follow-up and prognosis of patients with other etiologies of heart failure.^7,8^ Studies with NT-proBNP in CA have shown good diagnostic accuracy, including being part of the evaluation for the prognostic staging of the disease.^9,10^ In addition to diagnostic and prognostic evaluation, biomarkers can also be used to assess the therapeutic efficacy of these patients, especially in hematologic patients under chemotherapy, where they may be cardiotoxic.^4^

It is important to point out that cardiac amyloidosis is an increasingly frequent disease due to the aging of the population. However, this disease is still underdiagnosed, especially in less developed centers or where high-cost tests are not easily accessible to the population that uses public services. Therefore, it is necessary to organize diagnostic flowcharts that are more accessible to most of the population, and the measurement of biomarkers such as NT-proBNP is very useful in this scenario.
